# The high-level basis of body adaptation

**DOI:** 10.1098/rsos.172103

**Published:** 2018-06-06

**Authors:** Kevin R. Brooks, Colin W. G. Clifford, Richard J. Stevenson, Jonathan Mond, Ian D. Stephen

**Affiliations:** 1 Department of Psychology, Macquarie University, Sydney, New South Wales, Australia; 2 Perception in Action Research Centre (PARC), Faculty of Human Sciences, Macquarie University, Sydney, New South Wales, Australia; 3 ARC Centre of Excellence in Cognition and its Disorders, Macquarie University, Sydney, New South Wales, Australia; 4 School of Psychology, UNSW Sydney, Sydney, New South Wales 2052, Australia; 5 Translational Health Research Institute, University of Tasmania, Launceston, Tasmania, Australia; 6 Centre for Health Research, School of Medicine, Western Sydney University, Sydney, New South Wales, Australia

**Keywords:** adaptation, aftereffects, body size and shape misperception, high level, low level

## Abstract

Prolonged visual exposure, or ‘adaptation’, to thin (wide) bodies causes a perceptual aftereffect such that subsequently seen bodies appear wider (thinner) than they actually are. Here, we conducted two experiments investigating the effect of rotating the orientation of the test stimuli by 90° from that of the adaptor. Aftereffects were maximal when adapting and test bodies had the same orientation. When they differed, the axis of the perceived distortion changed with the orientation of the body. Experiment 1 demonstrated a 58% transfer of the aftereffect across orientations. Experiment 2 demonstrated an even greater degree of aftereffect transfer when the influence of low-level mechanisms was reduced further by using adaptation and test stimuli with different sizes. These results indicate that the body aftereffect is mediated primarily by high-level object-based processes, with low-level retinotopic mechanisms playing only a minor role. The influence of these low-level processes is further reduced when test stimuli differ in size from adaptation stimuli.

## 1. Background

In estimating the size and shape of their own bodies, many humans make consistent errors. Some, including sufferers of the eating disorder anorexia nervosa, erroneously perceive themselves to be much larger than they actually are [1–3], while others who are overweight or obese often perceive themselves to be of normal size [4,5]. Recent experimental studies have suggested that these examples of body size and shape misperception may be underpinned by the process of visual adaptation [6]. It has long been known that prolonged viewing of a stimulus can lead to perceptual aftereffects, such that subsequently seen images have an appearance that is opposite to the original stimulus [7]. For example, extended exposure (or ‘adaptation’) to downward motion can cause an aftereffect of illusory upward motion when the motion stops: the well-known motion aftereffect [8]. Conceivably, repeated and extended exposure to thin models in traditional and social media may yield a fattening visual aftereffect for those who consume such images, such that their own bodies viewed in the mirror may be misperceived as larger than they really are. On the other hand, individuals repeatedly exposed to large bodies—those of family members or friends for example—may experience a slimming aftereffect, leading them to view themselves as being thinner than they are in reality.

Adaptation causes perceptual aftereffects by inducing a change in the response properties of the neurons that are activated by the adaptation stimulus [9]. Extended exposure causes a recalibration of the relationship between stimulus parameters and neural activity, and hence a stimulus that would previously have elicited a particular pattern of activation now elicits responses that were previously associated with a different stimulus. By assessing the properties of the aftereffects, previous research has used adaptation as a non-invasive method of probing the neural basis of perception, leading some to refer to it as the ‘psychophysicist's microelectrode’ [10].

Some aftereffects, such as those arising from adaptation to motion, colour or the orientation of bars or edges, have been demonstrated to be ‘retinotopic’, i.e. their effects are restricted to the area of the retina that is exposed to the adaptation stimulus. This indicates that the neurons responsible for the perception of these ‘low-level’ properties are located at early stages of visual processing, where each neuron responds only to stimuli within a small region of the retina (i.e. its receptive field). Other aftereffects (e.g. those involving the identity of faces) can occur even when adaptation and test stimuli appear in different retinal regions [10,11]. This observation is consistent with the idea that facial identity is coded by cells in higher level areas with larger receptive fields.

Further evidence of the high-level nature of face aftereffects is provided by a study using adapting faces with horizontal distortions, presented tilted either at a +45° or −45° angle [12]. The resulting aftereffects were tested using faces either with the same orientation as the adaptor, or at right angles to it. If low-level mechanisms are responsible for encoding the geometry of the face, the aftereffect should be fixed with respect to the image on the retina, such that test images presented at 90° to the adaptor should show a vertical, rather than a horizontal distortion, relative to the face. However, if the aftereffect is a reflection of higher level mechanisms that encode the face as an object regardless of its retinal orientation, the distortion aftereffect should remain horizontal with respect to the test face at all orientations. In fact, the aftereffect did remain horizontal with respect to the face in all conditions, suggesting that the adaptation affected higher level neurons operating with an object-centred (rather than a retinal) frame of reference.

Although it is often assumed that body size and shape aftereffects are high-level effects, a rigorous test of this hypothesis has yet to be conducted. The first report of body size aftereffects used adaptation images that were uniformly expanded or contracted horizontally, shifting the perceived width of subsequently seen bodies in the opposite direction [13]. These image manipulations correspond to a decrease or an increase of the spatial frequency of vertical components of the image, and hence this result could reflect a simple low-level spatial frequency aftereffect [14,15]. While subsequent investigations have used more sophisticated representations of larger and smaller individuals [16–24], it is inevitable that images of heavier bodies will tend to include lower spatial frequencies, while lighter bodies will include higher spatial frequencies. As such, an explanation based on low-level spatial frequency adaptation cannot be definitively ruled out. The current study attempts to establish the relative contribution of high- and low-level processes in body adaptation in two experiments.

## 2. Experiment 1

**Figure 1.**
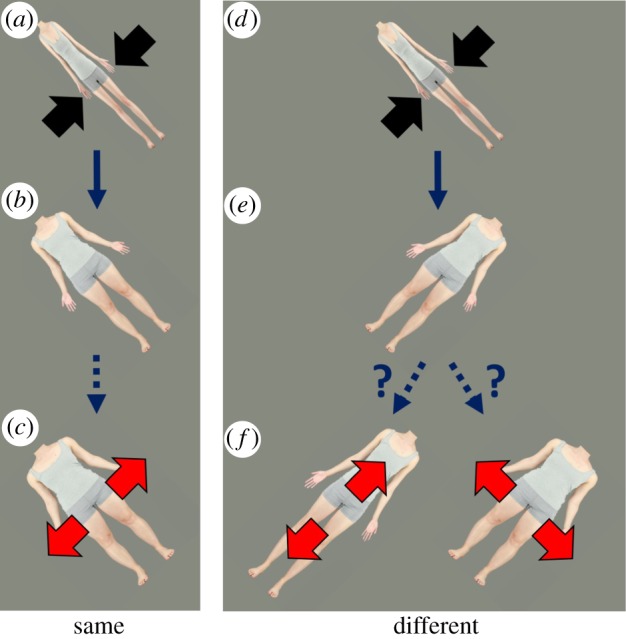
Outline of stimulus conditions and phenomenological predictions. For the ‘same’ relative orientation conditions (left column), prolonged exposure to the adaptation stimulus (*a*), in this case physically distorted to be thin, will cause an undistorted test stimulus (*b*) to appear perceptually wider (*c*). However, in the ‘different’ relative orientation conditions (right column), the same adaptation stimulus (*d*) could, in principle, make an undistorted test stimulus at right angles (*e*) either look taller (and hence narrower for its height) or wider (*f*), depending on whether the effect operates in a retinotopic or an object-centred frame of reference, respectively. Similar predictions can be made for conditions where adapting stimuli are physically distorted to be wide (not shown).

The current study adopts the ‘transfer across orientations’ approach [12] to establish the neural locus of body size and shape aftereffects. Using body stimuli that have been uniformly expanded or compressed along the transverse axis, we assess the magnitude and direction of the aftereffect that results when adaptation and test images are presented obliquely. Regardless of the locus of the adapted mechanism, we expect that when adapting and test stimuli are presented at the same orientation, exposure to a contracted adaptation stimulus will cause an unaltered stimulus to appear expanded as shown in figure 1 (left column). Consequently, the stimulus that actually appears normal will be one that has been contracted. This is reflected in a decreased ‘point of subjective normality’ (PSN) for width. Complementary effects are expected following adaptation to expanded body stimuli.

When the orientations of adaptation and test stimuli differ (figure 1, right column), low- and high-level perceptual mechanisms generate different predictions. If the mechanisms responsible for body aftereffects are high level, and operate using a body-centred frame of reference, the aftereffects caused by prolonged exposure to contracted images will again involve a decreased PSN, as is the case when adaptation and test stimulus orientations match. On the other hand, if low-level mechanisms with a retina-based frame of reference are at work, then for adaptation and test stimuli oriented at right angles to each other, an aftereffect caused by an adaptation stimulus contracted along the body's transverse axis will affect the test body along its longitudinal axis. This will make it appear taller, and hence, under the assumption that body width judgements are made relative to the height of the individual, an increase in width would be required to reach a normal appearance, leading to an increased PSN. These predictions are tested in two experiments.

### 2.1. Methods

#### 2.1.1. Participants

Twenty-seven female Caucasian students enrolled in an undergraduate psychology course at Macquarie University received course credit for their participation. Participants' ages ranged from 18 to 30 years (*M* = 19.4, s.d. = 2.6). All had normal or corrected-to-normal vision and gave written informed consent before agreeing to participate. All experiments were conducted in accordance with the Declaration of Helsinki and were approved by the Macquarie University Human Research Ethics Committee.

#### 2.1.2. Design

Employing a 2 × 2 mixed factorial design, this experiment included one between-subjects and one within-subjects independent variable. The between-subjects variable—‘adaptation condition’—had 2 levels: contracted (*N *= 13) and expanded (*N *= 14). The within-subjects variable—‘relative orientation’—had 2 levels: same and different. The dependent variable involved the stimulus width that appeared normal, i.e. the PSN. PSN was measured both before (baseline) and after adaptation, and the difference between the two scores was calculated for each individual for further analysis.

#### 2.1.3. Stimuli

We used 10 full-body photographs of female subjects (lighting controlled, neutral expression, frontal viewpoint, pose in anatomical position, tight-fitting grey shorts/singlet: see figure 1) selected from the original set described in [25]. Each of these 10 (and the manipulated images created from them), is referred to as a separate identity. Photographic subjects were between the ages of 18 and 27 (*M* = 21.2, s.d. = 3.6), with body mass indices in the normal range (21.4 to 22.4 kg m^−2^, *M* = 21.65, s.d. = 0.21) to ensure the consistency of form.

Adobe Photoshop CS6 was used to create experimental stimuli. As in previous studies [13], the heads were cropped out of the images to prevent explanations of our results based on face adaptation [12]. Images were then superimposed on a uniform grey background. For each original image (see electronic supplementary material, figure S1), we created 12 additional test stimuli varying in aspect ratio from −30% to +30% in 5% increments, and 2 adaptation images with aspect ratios manipulated to −50% and +50% compared to the original image. Each was formatted to a standard height of 500 pixels. For each observer, 2 of the 10 stimulus identities were randomly assigned to be used as test stimuli, while the other 8 were used as adaptation stimuli. Adaptation stimuli were presented either at a frontoparallel plane orientation of −45° or +45° (randomly assigned for each participant), while test stimuli were presented at both −45° and +45° in separate conditions for all participants. As we wished to assess the possibility that low-level adaptation might contribute to any resulting adaptation effect, both adaptation and test stimuli were the same size. All images were presented using Matlab^®^ version 7, operating Psychophysics Toolbox extensions [26] on a Dell P1130, 21^″^ colour monitor, viewed from a distance of 114 cm.

#### 2.1.4. Procedure

The experiment included a short practice phase followed by a baseline block prior to the adaptation block, and took approximately 20 min to complete. For baseline data collection, a ‘yes--no’ psychophysical procedure was used. In each trial, a body stimulus appeared on the screen for 1 s, followed by a grey screen and a brief tone to indicate that a response was required. Participants used a two-button mouse to indicate whether they thought the body appeared larger (wider) or smaller (thinner) than a normal, unmanipulated image. Participants were instructed to maintain their heads in an upright position throughout and were encouraged to respond as quickly and as accurately as possible, following which the next trial began after a 100 ms inter trial interval.

The level of stimulus width contraction/expansion of the test stimulus used in each trial was guided by a double interleaved 1-up-1-down adaptive staircase routine (see [22] for details). Beginning with ±50% steps, the step size was reduced by 15% after each reversal down to a minimum step size of 5%. Each staircase terminated after eight reversals at which point the mean distortion level of each participant's last six reversals was calculated to represent the PSN. Each staircase proceeded until it reached a maximum of 30 trials. If participants did not reach eight reversals, but gave consistent responses to the extreme test stimulus (−30% or +30%), the PSN was recorded as the appropriate extreme value. Two staircases, one starting at −30% and one at +30%, were randomly interleaved for each condition. Each participant's baseline PSN was calculated as the average of the PSNs from the two staircases.

Adaptation data collection followed the baseline block. This progressed in the same manner as baseline testing with the following exceptions. Testing began with a 120 s initial adaptation phase, during which participants were exposed to alternating images of the 8 adaptation identities (3 s each presentation: 5 repetitions of the entire sequence). In between each trial, 6 s of top up exposure, involving two of the adaptation identities selected at random, ensured that adaptation was maintained before the next test stimulus was presented. A brief 100 ms blank interval separated adaptation and test stimuli.

### 2.2. Results and discussion

**Figure 2.**
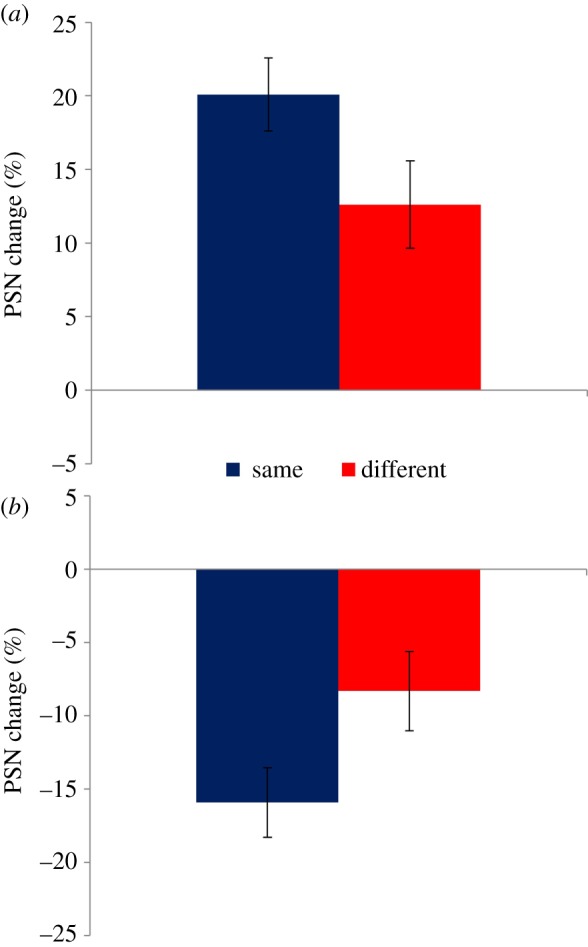
Data for experiment 1. (*a*) PSN change following adaptation to expanded stimuli. Subsequently seen stimuli appear thinner, resulting in a wider stimulus being perceived as normal, and a positive PSN for both relative orientation conditions. (*b*) PSN change following adaptation to contracted stimuli. Subsequently seen stimuli appear wider, resulting in a thinner stimulus being perceived as normal, and a negative PSN for both relative orientation conditions. In both (*a*) and (*b*), effects are larger when test patterns have the same orientation as the adaptor, compared to when orientations differ. Error bars represent ±1 s.e.m.

The change of PSN due to adaptation can be seen in figure 2. Informal observation suggests that, as expected, the PSN is consistently changed in the direction of the adapting stimulus. Following adaptation to expanded stimuli, wider figures appear normal (figure 2*a*), while adaptation to contracted stimuli causes thinner figures to appear normal (figure 2*b*). These effects have the same direction regardless of the relative orientation of adaptation and test stimuli. However, the magnitude of the effect appears larger when test and adaptation stimuli have the same orientation. These observations were confirmed by formal statistical analysis. In a set of four one-sample *t*-tests, mean PSN change values for all conditions differed significantly from zero (Expand Same: *t*_13_ = 8.050, *p *< 0.0005, *d *= 2.152; Expand Different: *t*_13_ = 4.254, *p *= 0.001, *d* = 1.137; Contract Same: *t*_12 _= −6.706, *p *< 0.0005, *d* = −1.860; Contract Different: *t*_12 _= −3.061, *p *= 0.01, *d* = −0.849), confirming the presence of aftereffects. As expected, there was no main effect of relative orientation in a 2 × 2 ANOVA. However, there was both a main effect of adaptation direction (*F*_1,25_ = 70.81; *p *< 0.0005; $\eta_{p}^{2}=0.739$) and a relative orientation × adaptation direction interaction (*F*_1,25_ = 21.128; *p *< 0.0005; $\eta_{p}^{2}=0.458$). Two-tailed repeated measures *t*-tests confirmed that PSNs for the ‘same’ relative orientation conditions were significantly more positive than ‘different’ PSNs for the expanded adaptation condition (*t*_13_ = 3.639, *p *= 0.003, *d *= 0.995), and significantly more negative for the contracted adaptation condition (*t*_12 _= −2.937, *p *= 0.012, *d* = −0.820).

The bias in the perception of body width following adaptation to either wide or narrow bodies, as shown in the ‘same’ relative orientation conditions, is consistent with many previous demonstrations of visual aftereffects of body size. However, the transfer of these effects between adapting and test bodies with different orientations has not previously been demonstrated. Given the substantial transfer of adaptation, such that both relative orientation conditions showed PSN shifts with the same direction, these results suggest that the processes mediating body shape and size aftereffects are predominantly high level in nature, operating within an object-centred, rather than a retinal frame of reference. However, it is noteworthy that aftereffects have a smaller magnitude when adaptation and test stimuli have different orientations. Taking the absolute value of scores for the expanded and contracted adaptation conditions, we can assess the overall transfer of the aftereffect across orientations, which stands at 58.3%. This is consistent with the idea of competition between high-level (same direction) and low-level (opposite direction) influences on perception, with the former ultimately showing greater influence.

## 3. Experiment 2

In experiment 1, the sizes (specifically, the heights) of adaptation and test stimuli were identical. In previous studies of high-level aftereffects, particularly face adaptation, efforts have been made to minimize the influence of low-level mechanisms by ensuring that the adaptation and test stimuli are different in size [27–34]. In experiment 2, we checked the effectiveness of this manipulation in the context of body adaptation. Specifically, we attempt to reduce the contribution of low-level aftereffects by changing the size of the test stimulus relative to the (fixed) size of the adaptation stimulus. If the introduction of differences in the relative height of adaptation and test stimuli is an effective method for reducing the influence of low-level effects, then the magnitude of PSN changes should be similar between relative orientation conditions. However, if stimulus size variation is ineffective at reducing low-level contributions, then the results of this experiment should show a close resemblance to those in experiment 1.

### 3.1. Methods

Methodological details for this experiment differed from those of experiment 1 only in the following respects. Thirty-two female Caucasian students enrolled in an undergraduate psychology course at Macquarie University received course credit for their participation. Participants' ages ranged from 17 to 30 years (*M* = 19.8, s.d. = 3.4). Although their aspect ratios were manipulated as in experiment 1, the test stimuli presented on each trial were isotropically scaled to appear to be a random height between 50% and 100% of the adaptation stimulus.

### 3.2. Results and discussion

**Figure 3.**
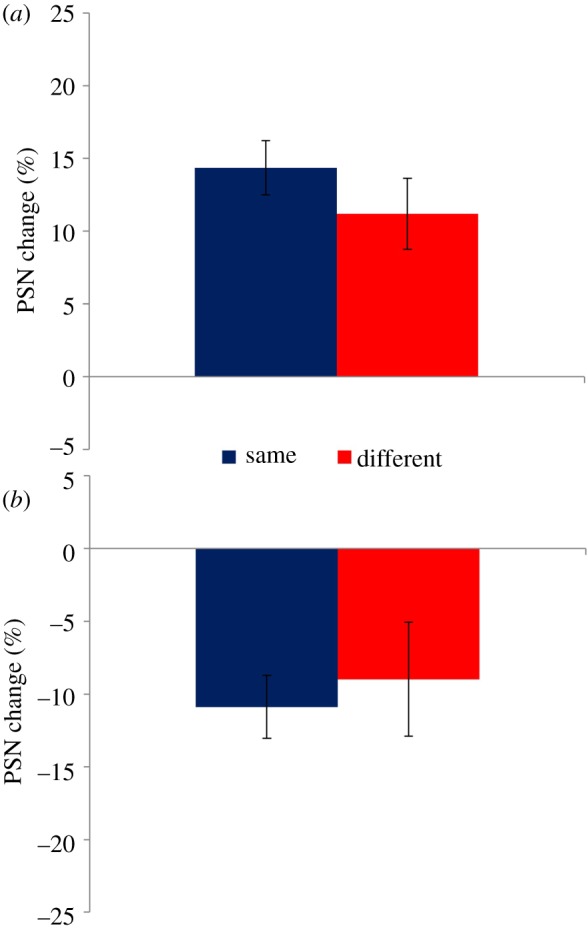
Data for experiment 2. (*a*) PSN change following adaptation to expanded stimuli. Subsequently seen stimuli appear thinner, resulting in a wider stimulus being perceived as normal, and a positive PSN for both relative orientation conditions. (*b*) PSN change following adaptation to contracted stimuli. Subsequently seen stimuli appear wider, resulting in a thinner stimulus being perceived as normal, and a negative PSN for both relative orientation conditions. In both (*a*) and (*b*), although effects appear to be marginally larger when test patterns have the same orientation as the adaptor, compared to when orientations differ, these differences are not statistically significant. Error bars represent ±1 s.e.m.

Figure 3 depicts the change of PSN due to adaptation for experiment 2. Here, as in experiment 1, the PSN is changed in the direction of the adapting stimulus for each condition. Adaptation to expanded stimuli (figure 3*a*) causes broader figures to appear normal, while narrower figures appear normal following adaptation to contracted stimuli (figure 3*b*). The relative orientation of adaptation and test stimuli has no bearing on the direction of these effects and appears to have little effect on their relative magnitude. Formal statistical analysis confirmed these observations. Four one-sample *t*-tests confirmed the presence of aftereffects in all conditions, as mean PSN change values differed significantly from zero (Expand Same: *t*_15_ = 7.747, *p *< 0.0005, *d* = 1.937; Expand Different: *t*_15_ = 4.571, *p *< 0.0005, *d* = 1.143; Contract Same: *t*_15 _= −5.011, *p *< 0.0005, *d* = −1.253; Contract Different: *t*_15 _= −2.294, *p *= 0.037, *d* = −0.573). As in experiment 1, there was no main effect of relative orientation in a 2 × 2 ANOVA, but a main effect of adaptation direction was apparent (*F*_1,30_ = 62.462; *p *< 0.0005; $\eta_{p}^{2}=0.676$). However, on this occasion the interaction between relative orientation × adaptation direction was not significant.

The similarities between the results of this experiment and those of experiment 1, in that body size and shape aftereffects transfer across different relative orientations, reinforce the conclusion that body adaptation is mediated largely by high-level processes. Furthermore, the differences between experiments, in that the magnitude of aftereffects was not significantly reduced when the orientations of adaptation and test stimuli were different (n.s. interaction), provide even greater support. Although a small difference between the magnitude of PSN changes is apparent in figure 3, with an aftereffect that is, on average, decreased to 80% of the value in the ‘same’ condition, this difference was not statistically significant. This suggests that the observed aftereffects are high level, object-centred effects with no evidence of competition from low level, retinotopic aftereffects.

## 4. General discussion

In two experiments, we have presented evidence that body size and shape aftereffects, even those arising from simple manipulations such as the uniform expansion or contraction of a body stimulus parallel to the transverse axis, are predominantly mediated by high-level processes. This confirms the implicit assumptions of many previous body adaptation studies. Furthermore, we have validated the common practice, inherited from face adaptation research, of varying the relative height of adaptation and test stimuli as a method of minimizing the contribution of low-level retinotopic adaptation effects.

Our study showed a transfer of adaptation across orientation of 58% and 80% in experiments 1 and 2, respectively. However, two caveats should be mentioned with respect to the accuracy of these values. Firstly, while the technique of randomly varying the size of the test stimuli was successful in reducing low-level contributions, this differs subtly from the technique used more commonly in face adaptation. Instead, a set difference in size between adaptation and test stimulus (often 25% or larger) is more usually employed and assumed to be sufficient to minimize the engagement of common low-level mechanisms by adaptor and test (although the magnitude of the size change necessary has not been empirically verified). For half of the trials in experiment 2, the size difference would have been larger than this value, up to 50%, but for the other half, the size difference would have been smaller, and in a very small number of trials, negligible. On these trials, low-level contributions are expected to be as large as in experiment 1. As such, it may be possible to reduce low-level contributions even further by ensuring that the sizes of adaptation and test stimuli are never similar. Secondly, although participants were instructed to keep their heads upright during viewing, and while the experimenter did not observe any head movements by participants, it remains possible that subtle head orientation shifts may have occurred. Such movements would be more likely to occur in the direction of the orientation of the stimuli, which would slightly increase the influence of the low-level effects in the ‘different’ orientation condition and hence inflate the observed extent of orientation transfer in both experiments.

Although transfer across orientation has not previously been demonstrated, one study did explicitly attempt to assess the possibility that low-level effects might contribute to body adaptation by testing the effect of adaptation to either thin or wide rectangles on subsequent body perception [18]. Here, the authors' interpretation that body-shape aftereffects cannot be explained by adaptation to low-level properties rests on the lack of bias in subsequent body judgements. However, there are other possible explanations for this null result, such as a lack of equivalence between body and rectangle adaptation stimuli (e.g. inappropriate rectangle widths), or differences in attention to the adapting stimuli (rectangles might conceivably be considered less engaging than body stimuli). Notably, there was no demonstration that adaptation to rectangles caused an aftereffect of perceived rectangle width. Our conclusions rest on the demonstration of positive effects, i.e. PSN changes for the ‘different’ relative orientation. This is a result that cannot be accounted for by low-level mechanisms, and that must therefore have a higher level explanation.

The observation that body aftereffects are high level in nature is of interest to researchers concerned with the neural representation of human form in visual areas, and the processes underpinning the normal perception of bodies. In addition, these observations support the contention that body size and shape adaptation may underlie certain real-world examples of disordered body image, such as those involving body size and shape misperception [6,35]. If perceptual distortions, such as those seen in eating disorders and related conditions, are the result of visual adaptation arising from repeated viewing of idealized images, the effects would need to be high level to transfer effectively between the pages of a magazine (or a smart phone screen) and the image reflected back at the observer in a mirror. A finding that the mechanisms of body adaptation were low level in nature would therefore have excluded such an explanation. Our findings, suggesting that body adaptation effects are indeed high level in nature, leave open the possibility that these fascinating and debilitating conditions may have perceptual aftereffects as their basis.

While low-level sources of body size and shape aftereffects can be effectively ruled out, the exact nature and locus of the effect have yet to be firmly established. Although observers who experience demonstrations of these effects subjectively report actually ‘seeing’ a distortion of body shape, it remains possible that a substantial proportion of the effects measured here and in other reports of body size and shape aftereffects [13,16–24,35] involve changes at the decision/criterion level, rather than a perceptual bias *per se* [36]. While both of these effects have relevance to real-world issues of body size and shape misperception, future experimental research may be informative in teasing them apart for the purpose of forming an improved understanding of normal and disordered processes of body perception.

## Supplementary Material

Supplementary Figure

## Supplementary Material

Data for Experiments 1 & 2

## Data accessibility

The datasets supporting this article have been uploaded as part of the electronic supplementary material.
