# Supplementary figures and images for: The high-level basis of body adaptation
Source: R Soc Open Sci. 2018 Jun 6;5(6):172103. doi: 10.1098/rsos.172103 (PMC6030264; doi:10.1098/rsos.172103)

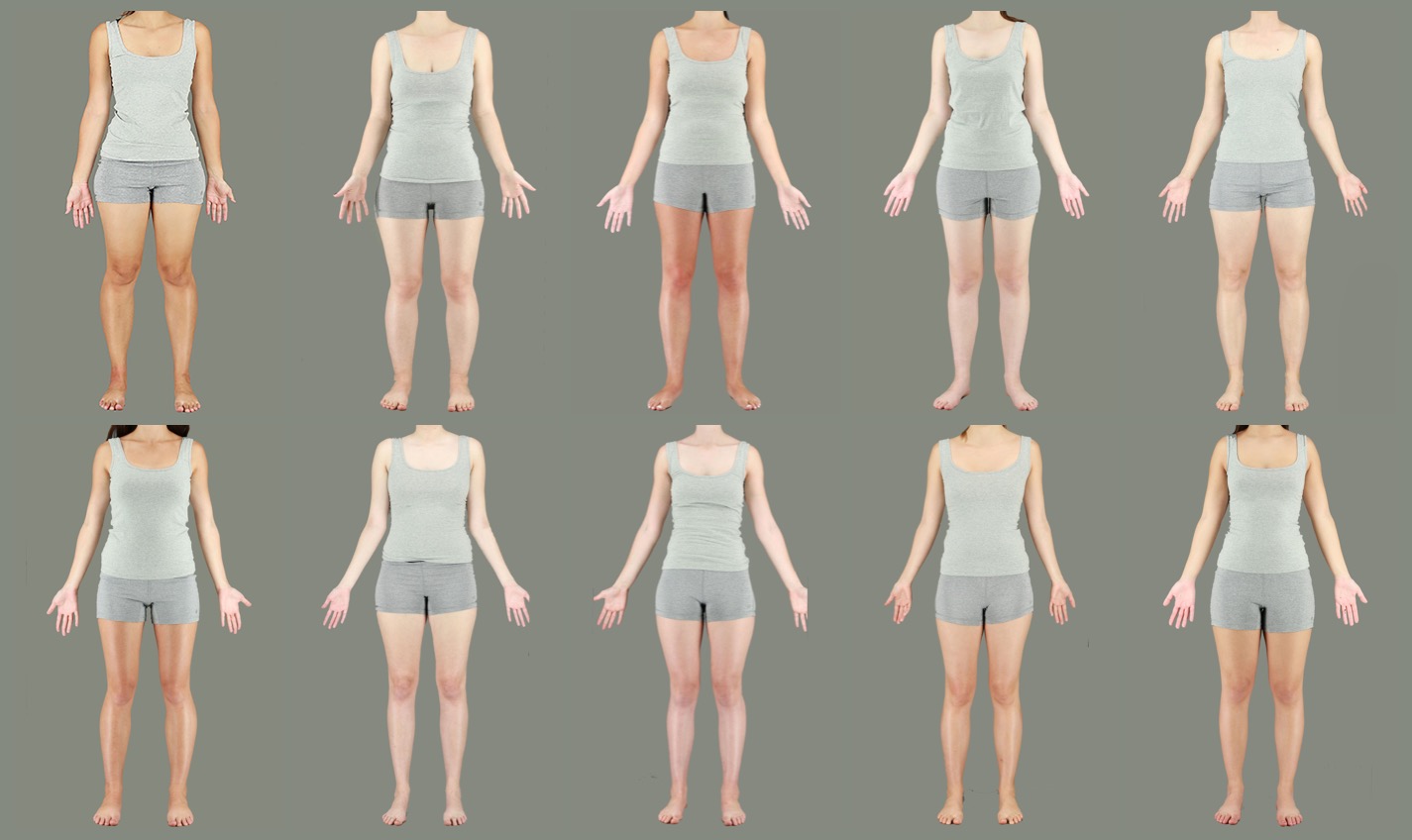

Supplement: Supplementary Figure [file rsos172103supp1.jpg]
